# Effects of time spent in pregnancy or brooding on immunocompetence

**DOI:** 10.1002/ece3.10764

**Published:** 2024-01-04

**Authors:** Vandana Revathi Venkateswaran, Chaitanya S. Gokhale, Marc Mangel, Sigrunn Eliassen

**Affiliations:** ^1^ Research Group for Theoretical Models of Eco‐evolutionary Dynamics, Department of Evolutionary Theory Max Planck Institute for Evolutionary Biology Plön Germany; ^2^ Department of Plant Biology, School of Integrative Biology University of Illinois at Urbana‐Champaign Urbana Illinois USA; ^3^ Institute of Marine Sciences and Department of Applied Mathematics University of California Santa Cruz California USA; ^4^ Theoretical Ecology Group, Department of Biological Sciences University of Bergen Bergen Norway

**Keywords:** immunocompetence, mathematical modeling, parental care, sexual dimorphism

## Abstract

Sexes of a species may show different characteristics beyond the differences in their sexual organs and such sexual dimorphism often occurs in the level of immune response when exposed to pathogens (immunocompetence). In general, females have increased longevity relative to males, which is associated with higher immunocompetence. However, males have higher immunocompetence in some species, such as pipefishes and seahorses. Experimental evidence suggests that this could be because males, rather than females, carry fertilized eggs to birth in these species. This observation suggests that an increase in immunocompetence may be related to the level of parental investment and not to a particular sex. We use state‐dependent life‐history theory to study optimal investment in offspring production relative to parent immunocompetence, varying the relative time that a parent spends in brooding or pregnancy within a breeding cycle. When offspring is dependent on a parent's survival for a large part of the breeding cycle, we predict higher investments in immunity and longer life expectancies.

## Graphical abstract

We hypothesize that (1) the amount of parental care has a direct effect on immunocompetence and (2) this effect should be seen while comparing individuals of the different sexes within a species or while comparing the major parental care providing sex between species. We explore this idea with a state‐dependent life‐history model to predict how an individual allocates its resources toward its own immunity while also investing in caring for its offspring in a way that it can maximize the fitness. The main result from our model is that an increase in investment toward immunocompetence is more pronounced in short‐lived species with longer brooding periods (in comparison to long‐lived species with shorter brooding or pregnancy periods). Our model also shows that in general, longer brooding times lead to more investment in immunity and hence higher life expectancy.

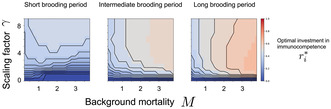

## 1 INTRODUCTION

Life‐history parameters such as body size, reproductive investment, and life expectancy vary across species, and within species, they often differ between males and females (Hedrick & Temeles, 1989; Nunn et al., 2008).

In this study, we focus on sex‐specific differences in immunocompetence, that is the ability of an individual to mount an immune response when exposed to pathogens, and we relate this to differences in parental care. Parental investment in offspring may consist of gamete production, brooding/pregnancy, and parental care (Trivers, 1972). Gamete production is common to both sexes, and the difference in gamete size (anisogamy) leads to the definition of the sexes: large gamete (egg) producers are referred to as females, and smaller gamete (sperm) producers are called males (Hayward & Gillooly, 2011; Liker et al., 2015; Parker et al., 1972). During the pregnancy or brooding period, offspring often depend mostly on one parent, most often the female but in some species, the male. Parental care can vary from nest building, egg brooding, and nuptial gifts, to caring for the young that can be performed by one or both parents (Smith, 1977; Wade & Shuster, 2002). Survival of the parent performing brooding, pregnancy, or other forms of parental care is often crucial for offspring success. Experimental results across diverse taxa show that increased time spent in parental investment is associated with higher investments in immunocompetence (Keller et al., 2017; Lin et al., 2016; Peck et al., 2016; Roth et al., 2011). This suggests that the sex investing more time in brooding and parental care may benefit from investing in immunocompetence to increase survival (Nunn et al., 2008). The benefit of parental investment for an offspring partly depends on the reserves allocated per time, and the time that a parent is able to provide those reserves. For instance, in mammals females tend to have increased longevity that comes with higher immunocompetence, which could be because female mammals go through pregnancy, that is periods of gestation (Forbes, 2007; May, 2007; Rolff, 2002).

The males undergo pregnancy and brooding in many pipefishes (such as *Syngnathus typhle*). A male receives eggs in its brood pouch, where the eggs are fertilized and nurtured (Wilson et al., 2003). Experimental studies show that males of these species have a higher immunocompetence than females (Roth et al., 2011). In another pipefish species (*Nerophis ophidion*), where the males perform only brooding, there is no profound sexual dimorphism in immunocompetence. In species of the genus *Hippocampus* commonly known as seahorses, males perform mating competition, pregnancy, and brooding. Studies on these species (e.g., Lin et al., 2016; Vincent et al., 1992) suggest an increased immunocompetence among males due to their higher parental investment. These results suggest that different levels of immunocompetence between species are based on the amount of parental investment, regardless of the sex that performs the investing. The evolution of sex‐specific differences in the amount of parental investment (Alonzo & Klug, 2012; Liker et al., 2015; Lonstein & De Vries, 2000; Trivers, 1972) and mating competition through ornamentations (Darwin, 1871; De Lisle, 2019; Jones et al., 2000) have been studied experimentally and theoretically. These studies have shown sexual dimorphism in immunocompetence, that is sexual immune dimorphism (Forbes, 2007; Nunn et al., 2008; Restif & Amos, 2010; Stoehr & Kokko, 2006). According to the immunocompetence handicap hypothesis (ICHH), investments toward elaborate secondary sexual traits and success in sperm competition have a trade‐off with investment in male immunocompetence (Folstad & Karter, 1992). Nunn et al. (2008) presented a means to study sexual immune dimorphism independently of the ICHH using the fact that there is a lack of testosterone in insects. But here too, they found a female bias in immunocompetence, showing that ICHH lacks generic empirical support. A model by Stoehr and Kokko (2006) highlighted that (1) longevity is typically more important to female fitness compared to male fitness and (2) the benefit from investing in immunocompetence is an increase in longevity. Stoehr and Kokko (2006) and Forbes (2007) also showed that the male immunocompetence is lower than that of females when the above two conditions are satisfied. However, Stoehr and Kokko (2006) also showed that sex differences in parasitic impact might cause males to invest more into immunocompetence. Medley (2002) predicted an increased investment in immunocompetence when the parasitic impact increases. However, these models had a different focus where they did not include parental investment.

Resource allocation has been observed across the tree of life for various life‐history traits (Antonovics, 1980; Rauw, 2012; Schärer & Janicke, 2009; Schütz et al., 2002). Here, we focus on parental investment and use state‐dependent life‐history theory (Clark & Mangel, 2000; Houston & McNamara, 1999; Mangel & Clark, 1988), to model parental investment and immunocompetence as the traits of interest. Current reproductive investment would often reduce the amount of reserves or decrease survival probability to the next breeding opportunity. We studied the trade‐offs between reserves allocated to these traits in such a way that they maximize an individual's expected reproductive success, determined by survival and reproduction. We thus elucidate the patterns of investment in reproduction and immunocompetence and their dependence upon specifics of the biological system and ecology.

## 2 METHODS

Organisms constantly face trade‐offs in resource allocation toward various traits to survive and reproduce successfully (Contreras‐Garduño et al., 2006; Lin et al., 2016; Loiseau et al., 2008; Mangel & Heimpel, 1998; Peck et al., 2016; Verhulst et al., 2005; Wedell & Karlsson, 2003). Individuals maximize fitness by allocating optimal amounts of reserves toward various life‐history activities and states. We denote by $R\left(t\right)$ the level of reserves used for reproduction and immunocompetence at the start of a discrete time period t, with $0<R\left(t\right)\leq R_{\max}=1$ (maximum value chosen for modeling convenience). These reserves are either allocated to improve the immunity/immunocompetence of the parent or to parental effort for producing offspring. We shall denote the reserves allocated toward immunity and offspring production to be $r_{i}$ and $r_{o}$, respectively. Thus $R\left(t\right)=r$, $r_{i}+r_{o}\leq r$.

### 2.1 Current reproduction

We assume that the number of offspring produced by a parent $\Phi \left(r_{o}\right)$ is a concave function of the amount of reserves allocated to investment in offspring production and write it as,

(1) $$\Phi \left(r_{o}\right)=\Phi_{\max}\cdot {r_{o}}^{\alpha },$$

where $\Phi_{\max}$ is the maximum number of offspring produced (when $r_{o}=1$) and 0<α<1 is a shape parameter. For the results presented in this study, $r_{o}=r-r_{i}$ and r is the total reserves at that time. Thus Equation 1 can also be written as,

(2) $$\Phi \left(r_{i}\right)=\Phi_{\max}\cdot {\left(r-r_{i}\right)}^{\alpha }.$$

The current reproductive success depends on both the reserves allocated toward investment in offspring production and the parent's survival (i.e., investment toward the parent's immunity) because we are considering parents that undergo brooding or pregnancy. We let M denote the background rate of natural mortality. If an individual does not allocate resources to immunocompetence, then the probability that it survives the current period is $e^{-M}$. Let us denote f as the fraction of time period spent in parental investment, that is, the relative length of the time spent in parental investment without which the offspring will not survive. For example, the relative fraction of time that offspring are dependent on a parent (f) would be very low for free‐spawning females and males. At the other end of the spectrum, f could be close to 1 for female bowhead whales because they spend all their reproductive time in parental care. We can also make comparisons within species to look at the effect of f on sexual immune dimorphism. This would be a situation where f would be high for the sex that does more parental investment and low for the other sex. Female mammals would for instance have relatively high f values in the same way as male seahorses, compared to their partners.

**FIGURE 1:**
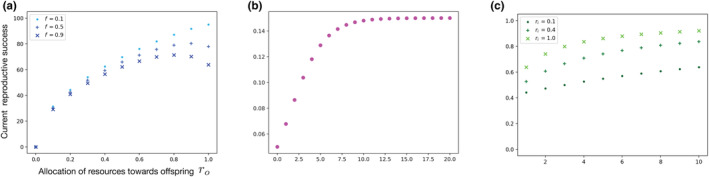
(a) The function describing current reproductive success, $\Phi_{\max}\cdot {\left(r_{o}\right)}^{\alpha }\cdot S_{\text{current}}\left(r-r_{o}\right)$ as a function of reserves allocated toward parental effort for producing offspring $r_{o}$ for different values of the relative fraction of time between breeding events spent in pregnancy or brooding (f). The value $\Phi_{\max}$ is the maximum number of offspring that can be produced. Here, α=0.5 and $\Phi_{\max}=100$. (b) Gain in reserves between each breeding event $g\left(t\right)$ initially increases with time t. Here, $g\left(t\right)=\frac{0.3}{2+4e-0.5t}$. We let $g\left(t\right)$ saturate in time to capture the notion of growth toward an asymptotic size after maturity. (c) Survival in current reproductive season ($S_{\text{current}}$) with varying γ for different values of $r_{i}$; for f=0.9 and M=1.0.

If $r_{i}$ is the investment in the parent's immunity, we assume that the probability of the parent's survival in the current reproductive period is $S_{\text{current}}\left(r_{i}\right)=e^{-\mathrm{Mf}/\left(1+\gamma r_{i}\right)}$, where γ scales the effect of investment in immunocompetence ($r_{i}$) on survival (see Figure 1c and Table 1). A high value of γ means that the individual has a good immune defense because it can increase survival with a small investment, while a small value of γ implies that significant investment is needed to improve survival. In fact, even when the background mortality is a high value like M=10, if f=0.1 and γ is big enough, then survival can become higher. Low and high values of γ could denote species with high and low ability to “fight” pathogens. Species with high γ would be individuals with good immune system and thus, they do not need to invest more toward immunocompetence. The fraction of living individuals at every time is also higher in species with high γ as compared to species with a lower γ value. Another interesting aspect to be mentioned here is that, gamma could also be interpreted as a reflection of the environment in which an individual lives. For example, individuals in an environment with many parasites, in which mortality depends strongly on immunocompetence, could have a higher gamma than individuals in an environment where parasites are absent and thus, immunocompetence would have little effect on mortality. Since we are modeling reproductive systems with brooding or pregnancy, we assume that offspring survive only if the parent that provides care survives the current reproductive period. The reproductive success would then be described by the following expression, illustrated in Figure 1a.

(3) $$\Phi \left(r_{o}\right)\cdot S_{\text{current}}\left(r_{i}\right).$$

**TABLE 1:** A list and summary of parameters used in the deterministic and stochastic model.

Parameter	Definition
$R\left(t\right),r$	Reserves at time t and a specific value for it
$g\left(t\right)$	Gain in reserves after each reproductive period
$R_{\max}=1.0$	Maximum value of total reserves
$r_{o}$	Reserves allocated toward parental effort for producing offspring
$\Phi_{\max}$	Maximum offspring produced, that is number of offspring produced when $r_{o}=1.0$
$\Phi \left(r_{o}\right)=\Phi_{\max}\cdot {r_{o}}^{\alpha }$	Offspring produced
$r_{i}$	Immune reserves allocated toward the parent's survival in a reproductive season
f	Relative fraction of time between breeding events spent in pregnancy or brooding
M	Background mortality
γ	Scaling factor for the effect of investment in immunocompetence ($r_{i}$) on survival
$S_{\text{current}}\left(r_{i}\right)=e^{\frac{-\mathrm{Mf}}{1+\gamma r_{i}}}$	Probability that the parent survives in the current reproductive season
$\Phi \left(r_{o}\right)\cdot S_{\text{current}}\left(r_{i}\right)$	Current increment in lifetime reproductive success
$S_{\text{future}}\left(r_{i}\right)=e^{\frac{-M}{1+\gamma r_{i}}}$	Probability of survival to next reproductive season
$W\left(r,t\right)$	Fitness function (the maximum accumulated lifetime reproductive success) given that $R\left(t\right)=r$
$\bar{g}$ and σ	Approximate mean and standard deviation in gain in reserves in a fluctuating (stochastic) environment
$g_{n}$ where n=1,2,…,N	The value of reserve gain that an individual k encounters or receives, where N is the number of intervals between the minimum and maximum values of the gain
$p\left(g_{n}\right)=\frac{e^{\frac{-{\left(g_{n}-\bar{g}\right)}^{2}}{2\sigma^{2}}}}{\sum_{g_{n}=0}^{N}e^{\frac{-{\left(g_{n}-\bar{g}\right)}^{2}}{2\sigma^{2}}}}$	Probability of obtaining $g_{n}$

### 2.2 Deterministic resource dynamics for semelparous life‐history

Since $r_{i}=r-r_{o}$, the equation for reproductive success (Equation 3) can also be written as $\Phi_{\max}\cdot {\left(r-r_{i}\right)}^{\alpha }\cdot S_{\text{current}}\left(r_{i}\right)$. For a semelparous individual that has just one reproductive season throughout its lifetime, which is the current season, the reproductive fitness will be written as,

(4) $$W\left(r|r_{i}\right)=\Phi_{\max}\cdot {\left(r-r_{i}\right)}^{\alpha }\cdot e^{-\frac{M\cdot f}{1+\gamma \cdot r_{i}}}.$$

The solution of the optimal investment in the parent's immune response ($r_{i}^{*}$) can be obtained from Equation 4 to be

(5) $$r_{i}^{*}=\frac{-\left(2+k\right)\pm \sqrt{\left({\left(2+k\right)}^{2}-4\cdot \left(1-\left(r\cdot k\cdot \gamma \right)\right)\right)}}{2\cdot \gamma },$$

where $k=\frac{M\cdot f}{\alpha }$. This ratio k determines the range of possible values for the parameters γ and M for $r_{i}^{*}$ to be a positive real solution (detailed derivations and calculations in Appendix S1). For $r_{i}^{*}$ to be finite and positive, that is for Equation 5 to give a positive solution, we need γ>0, and

(6) $$r\cdot \left[\frac{M\cdot f\cdot \gamma }{\alpha }\right]\geq 1.$$

Details of obtaining the above inequality are also in the Appendix S1. In Equation 6, if r=1, and say, for instance, f=0.5 (an intermediate value for the relative fraction of time between breeding events spent in pregnancy or brooding f), and α=0.5 as in all the figures in this paper, then,

(7) $$M\geq \frac{1}{\gamma }.$$

**FIGURE 2:**
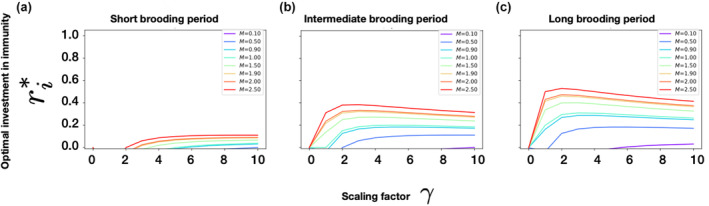
Analysis of the relative fraction of time between breeding events spent in pregnancy or brooding (f), background mortality M, and the scaling factor γ on the optimal investment toward the parent's immunity $r_{i}^{*}$. Here, f equals 0.1, 0.5, and 0.9 for short, intermediate, and long relative fractions of time between breeding events spent in pregnancy or brooding, respectively. The different values of f could be used to compare two different species and also compare the two sexes within a species, that is, a short relative fraction of time between breeding events spent in pregnancy or brooding (short f) can be used to denote male lions while a longer f would be the female lions.

Therefore, we observe that $r_{i}^{*}=0$, when M⋅γ=1. The solution $r_{i}^{*}\geq 0$ for M=0.1 only when γ≥10. Likewise, $r_{i}^{*}\geq 0$ for M=0.5 only if γ≥2, and so on, as shown in Figure 2 (please refer to Figures S1 and S2 in the Appendix S1 for detailed analyses).

Thus, we see how in a semelparous case, for a specific background mortality M, as f increases, $r_{i}^{*}$ also rises with increasing γ (scaling factor on the effect of invest in immunity toward survival). This is because the individuals with longer brooding periods need to invest more to their immunity to stay alive for that entire brooding period while the individuals with low brooding periods do not have that pressure. However, we notice that after a certain threshold value of background mortality M, an increase in the scaling factor γ leads to a decrease in $r_{i}^{*}$. This is due to the fact that beyond a certain M, there is no more point in the parent investing more toward its own immunity $r_{i}^{*}$. For there is only one reproductive season, and the parent should also focus on investment toward offspring production. This way the individual ensures maximum fitness from that season because this will be the lifetime reproductive fitness for a semelparous individual. Therefore, the parent relies more on its γ. We have also shown that there is also a range of M and γ for which a real solution for $r_{i}^{*}$ cannot exist (i.e., for certain combinations, $r_{i}^{*}$ will be nonnegative and since we cannot have a negative value for optimal allocation those results are not valid and hence, not plotted; please see Appendix S1 for the full details). Thus, under the specifications of this model, there cannot exist species with certain combinations of M and γ. For instance, if a species has a very low f, we see how high its M must also be (for $r_{i}^{*}$ to be non‐negative). This condition is consistent with semelparous short‐lived species with a short brooding period, such as mayflies. Thus our method can capture some possible patterns of species life histories in nature.

### 2.3 Iteroparous life‐history

In the deterministic iteroparous or multiple brooding season cases, the reserve gain is a function of time given by $g\left(t\right)$. This deterministic gain in reserves can be a function as shown in Figure 1b. In the next section, we shall consider a stochastic case where we let G denote the random variable characterizing stochastic reserves gain. If $R\left(t\right)=r$ and the allocations to offspring and immune function are $r_{o}$ and $r_{i}$, respectively, the reserves at the start of the next period, denoted by $r^{\prime }\left(r,r_{o},r_{i},t+1\right)$ are

(8) $$r^{\prime }\left(r,r_{o},r_{i},t+1\right)=r-r_{o}-r_{i}+g\left(t\right).$$

To calculate the expected lifetime reproductive success, one needs to know the future and current reproductive success. We use state‐dependent life‐history theory implemented by stochastic dynamic programming (SDP) (Mangel & Clark, 1988) to investigate the optimal allocation toward immunocompetence and reproduction. As a fitness metric, we use accumulated lifetime reproductive success (Roff, 1993; Stearns, 1992). We let $W\left(r,t\right)$ denote the maximum accumulated lifetime reproductive success from time t onward, given that the $R\left(t\right)=r$. We refer to $W\left(r,t\right)$ as fitness at time t for r amount of reserves. Example: expected lifetime reproductive success starting with reserve level $R_{0}$ is $W\left(R_{0},1\right)$.

**FIGURE 3:**
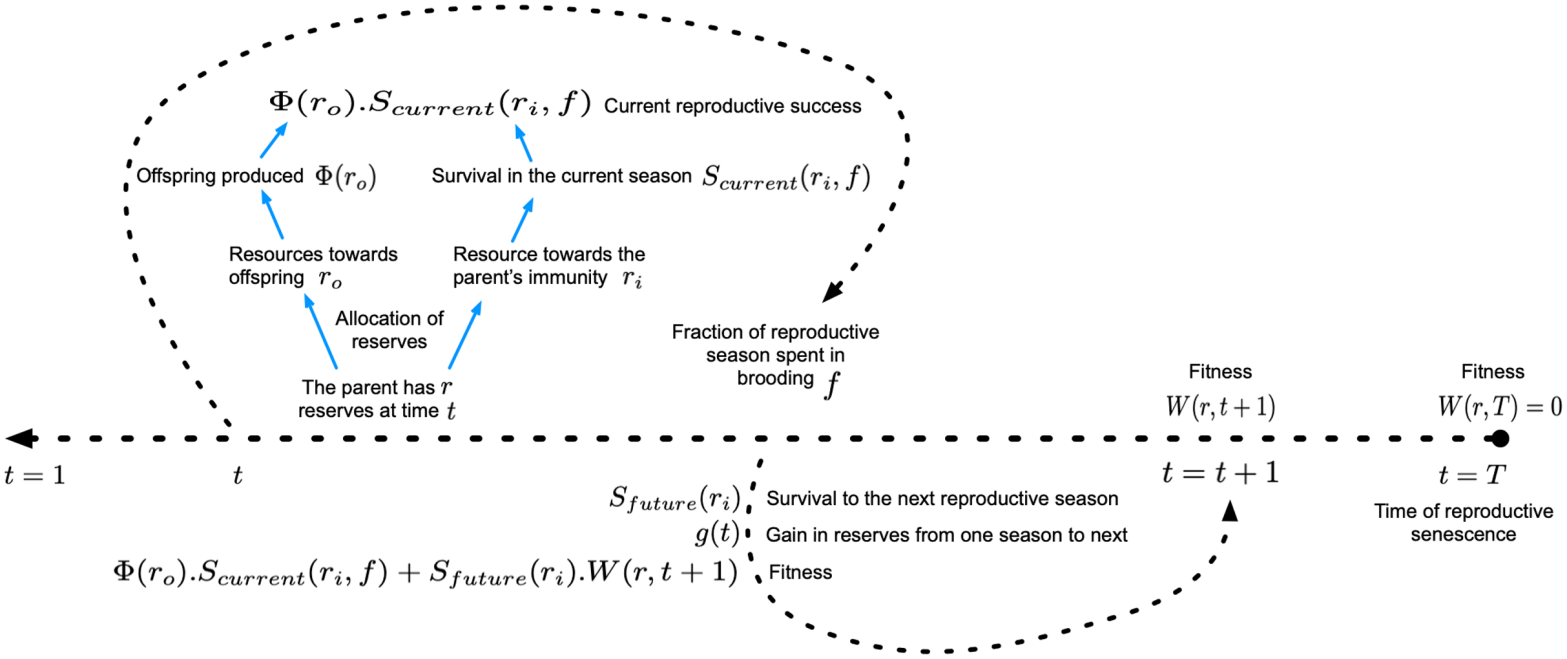
Allocation of a parent's reserves and the backward routine process. This backward method allows one to generate the rules of reserves allocation. The reserves allocated toward parental effort for producing offspring $r_{o}$, the parent's own immunity $r_{i}$, and the relative fraction of time between breeding events spent in pregnancy or brooding (f) between one reproductive season to the next (i.e., between t and t+1) determine the current reproductive success. Survival of this parent to the next season ($S_{\text{future}}$) depends on the amount of reserves for immunity it allocates toward future survival. This survival to the future seasons determines the future reproductive success. The expected lifetime reproductive success is given by $W\left(r,t\right)$ at time t. We go through a backward iteration since we start at a certain end time point and go backwards to find the values of $r_{o}$ and $r_{i}$ that maximize the fitness at every time t for every r value. This gives us a rulebook with values for the optimal reserves allocated toward offspring production ($r_{o}^{*}$) and parent's immunity ($r_{i}^{*}$), at every time t for every r value.

We find the allocations to the parent's immunocompetence $r_{i}$ and parental effort toward offspring production $r_{o}$ that maximize $W\left(r,t\right)$ for every value of r and t as follows. First, we assume reproductive senescence occurs at time T, after which no reproductive success is accumulated so that $W\left(r,T\right)=0$ for all values of r. For previous times, $W\left(r,t\right)$ satisfies the following equation of SDP (e.g., Chapter 4 in Clark & Mangel, 2000; Chapter 4 in Mangel & Clark, 1988). The maximum accumulated lifetime reproductive success $W\left(r,t\right)$ given $R\left(t\right)=r$ then satisfies the iteration

(9) $$W\left(r,t\right)=\max_{r_{i},r_{o}}\left[\Phi \left(r_{o}\right)\cdot S_{\text{current}}\left(r_{i}\right)+S_{\text{future}}\left(r_{i}\right)\cdot W(r^{\prime }(r,r_{o},r_{i},t+1),t+1))\right],$$

where $S_{\text{future}}\left(r_{i}\right)=e^{-M/\left(1+\gamma r_{i}\right)}$ is the probability that the parent survives to the next reproductive period. The first term on the right‐hand side of Equation 9 is current reproductive success, given $R\left(t\right)=r$, and the second term is the expected future reproductive success given the change in reserves. Figure 3 illustrates this entire SDP method used in our model. We summarize the variables and parameters in the model in Table 1. In the course of solving Equation 9, we determine the optimal values of investment, $r_{o}^{*}\left(r,t\right)$ and $r_{i}^{*}\left(r,t\right)$ for every possible value of r and t. We solve Equation 9, backwards from end time T to a starting time to get the optimal values for $\left(r_{o},r_{i}\right)$ from r that the individual would allocate for all the times. This gives us a rulebook with values of $r_{o}^{*}\left(r,t\right)$ and $r_{i}^{*}\left(r,t\right)$.

**FIGURE 4:**
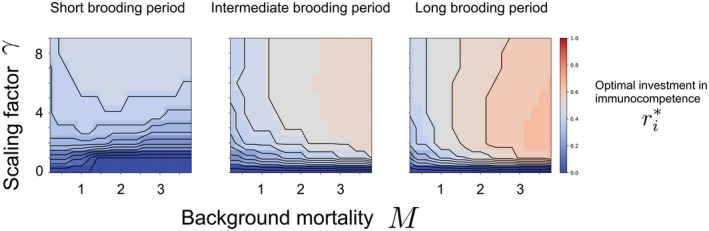
Optimal investment in the parent's immunocompetence ($r_{i}^{*}$) as a function of background mortality (M), scaling factor for the effect of investment in immunocompetence on survival (γ) for different values for the relative fraction of time between breeding events spent in pregnancy or brooding (f). These optimal values were calculated from the state‐dependent dynamics programming backward method. These results are for $R\left(t=11\right)=R_{\max}$, where $R_{\max}=1.0$ is the maximum value of total reserves as mentioned in Table 1. Here, we denote short brooding period, intermediate brooding period, and long brooding period for f=0.1, f=0.5, and f=0.9, respectively. From the plots for optimal investment in immunity, we see that in general, more reserves are allocated toward immunocompetence as the relative fraction of time between breeding events spent in pregnancy or brooding (f) increases. Here, the optimal investment in reserves allocated toward parental effort for producing offspring ($r_{o}^{*}$) would just be $R_{\max}-r_{i}^{*}$.

Figure 4 shows the range of results that the backward method can produce. With M and γ fixed, more reserves are allocated toward immunocompetence as the relative fraction of time between breeding events spent in pregnancy or brooding (f) increases. This is so because the parent's survival throughout the brooding period is essential for the offspring's survival and production. Holding γ constant, we explore changes in background mortality M. First, when the breeding period is short (f=0.1), for a fixed value of γ (say γ=5), the optimal investment in immunocompetence ($r_{i}^{*}$) initially increases for increasing values of background mortality and then there is a dip before it increases again. In the analysis that we showed earlier, we see this effect of increasing positive value of $r_{i}^{*}$ with increase in M and γ after a certain threshold M. This is because when mortality is very low, individuals are anyway likely to survive, even without much allocation of reserves to immunocompetence ($r_{i}$). However, there is also another threshold value for M after which $r_{i}$ starts to decrease, and this is more drastic for short brooding period (low f). Since background mortality M is so high, the allocation toward $r_{i}$ becomes unimportant since the individuals are unlikely to survive regardless. This is why we see a dip in that curve.

Our analysis showed that for a short relative fraction of time between breeding events spent in pregnancy or brooding, when M and γ increase, the likelihood of surviving to the next breeding season will be small. This trend will be the same for intermediate and long brooding periods (f=0.5 and f=0.9, respectively). Hence, for higher f, the individual needs to invest more toward its survival, that is immunity in the current breeding season. If not, it will have a reduced reproductive output. This is why we see less allocation to $r_{o}$ for longer f as compared to short f. From the plots shown for optimal investment in offspring, we see that, in general, more reserves are allocated toward immunity as the relative fraction of time between breeding events spent in pregnancy or brooding (f) increases. This is because the survival of the parent through the brooding period is absolutely necessary for the offspring survival and production, and therefore the individuals have to focus more toward immunity because if not, then they may risk not surviving to take care of the offspring. Think of pipefish or seahorse males who need to survive to take care of the fertilized eggs within their brooding pouch, or a lioness who needs to invest more toward her survival as well for tending to her offspring in her womb and also taking care of the cubs for a certain period after parturition. In these scenarios, parental care is mandatory from parturition till post‐parturition, without which the offspring would die. Thus, in these cases, the parent's survival is crucial, and reserves toward investment in offspring production alone would not suffice.

**FIGURE 5:**
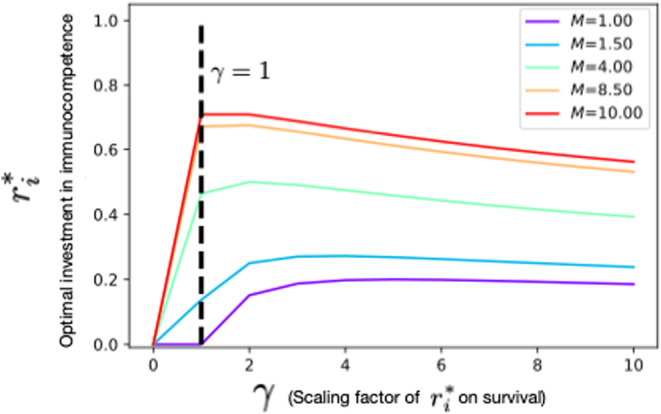
Effect of the scaling factor γ and background mortality M on the optimal investment in immunity $r_{i}^{*}$. In this simple analysis, we consider a semelparous case where an individual has only one reproductive episode before death. The lifetime reproductive fitness of semelparous individuals would just be the current reproductive success $\Phi_{\max}\cdot {\left(r-r_{i}\right)}^{\alpha }\cdot S_{\text{current}}\left(r_{i}\right)$ also written as $\Phi_{\max}\cdot {\left(r-r_{i}\right)}^{\alpha }\cdot e^{-\mathrm{Mf}/\left(1+\gamma r_{i}\right)}$. If we keep constant values for f and α, we obtain the effect of γ and background mortality M on the optimal investment in immunity $r_{i}^{*}$. As this plot shows, if for a certain γ (for example, γ=1 as shown here) we increase M, then $r_{i}^{*}$ increases, but only after a certain threshold value of γ. This is because of the condition M.γ≥1 that was derived in Equation 7 for $r_{i}^{*}$ to be a nonnegative solution. Keeping M constant and increasing γ, then until a certain threshold value of γ, the parameter $r_{i}^{*}$ also increases. Here, f=0.5 and α=0.5. After that threshold, $r_{i}^{*}$ starts to decrease with γ. See Appendix S1 for details and derivations.

Finally, we explore the impact of holding M constant and changing γ. In Figure 5, we hold the relative fraction of time between breeding events spent in pregnancy or brooding as f=0.5, and show a detailed analysis of the effect of γ and M on $r_{i}^{*}$. From this analysis, we find that, for a fixed M and increasing γ, until a specific threshold value of γ, the parameter $r_{i}^{*}$ also increases. After that threshold, $r_{i}^{*}$ starts to decrease. This is because high γ takes care of the survival and the individual does not need to invest more toward its immunity. We also find that when γ is fixed and M increases, then $r_{i}^{*}$ also increases. This is because when γ is constant or cannot change, with increase in background mortality, the benefit of investing toward immunity also rises to be able to survive that breeding/gestation period, and also to survive to the next reproductive season. Thus, for certain values of the relative fraction of time between breeding events spent in pregnancy or brooding (f), the parent's $r_{i}^{*}$ may either decrease or increase depending on the values of M and γ. This effect will increase with increasing f (see Appendix S1 for the full details and derivations) since the individual needs to invest more toward its immunity when the brooding or gestation periods are longer.

### 2.4 Stochastic resource dynamics

In the previous section, we assumed that the reserves increased deterministically between breeding events. However, nature is stochastic and therefore, to obtain realistic results, we now let the gain in reserves be a random variable. We model the scenario where an individual first encounters a site and then allocates its reserves based on knowledge of the gain at that site (e.g., stickleback males first choose a good territory to make their nests, after which they spawn with females and fertilize eggs; Rushbrook et al., 2008). In an alternative scenario, individuals allocate the reserves before encountering the site (e.g., some insects and amphibians who fertilize the eggs before and then enter a site to lay their larvae; Jaenike, 1978).

We let $\tilde{G}$ denote a random variable corresponding to the reserve gain at the end of the breeding period and assume that all breeding sites share the same distribution for $\tilde{G}$. In addition, we assume that $g_{\min}\leq \tilde{G}\leq g_{\max}$.

**FIGURE 6:**
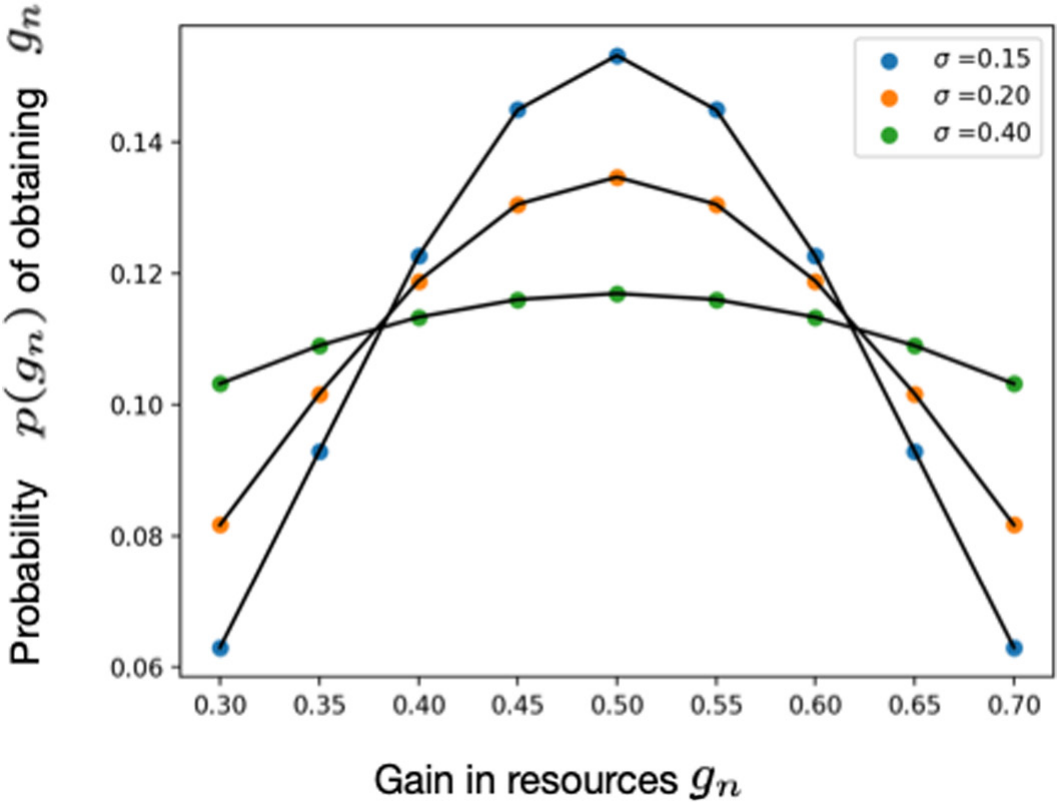
Stochastic gain in reserves for varying variance σ. Here, $g_{n}$ is a specific realization of the random process $\tilde{G}$ characterizing gain at the end of a reproductive period. We use a discrete Gaussian distribution so that Pr[$\tilde{G}=g_{n}$] = $p\left(g_{n}\right)=\frac{e^{\frac{-{\left(g_{n}-\bar{g}\right)}^{2}}{2\sigma^{2}}}}{\sum_{n=0}^{N}e^{\frac{-{\left(g_{n}-\bar{g}\right)}^{2}}{2\sigma^{2}}}}$. See main article text for further details.

We divide the range between the minimum and maximum values of the gain into N intervals, let $g_{n}=g_{\min}+\left(n/N\right)\left(g_{\max}-g_{\min}\right)$, where n ranges from 0 to N, denote the probability that $\tilde{G}=g_{n}$ by $p\left(g_{n}\right)$, and assume a discrete Gaussian distribution

(10) $$p\left(g_{n}\right)=\frac{e^{\frac{-{\left(g_{n}-\bar{g}\right)}^{2}}{2\sigma^{2}}}}{\sum_{n=0}^{N}e^{\frac{-{\left(g_{n}-\bar{g}\right)}^{2}}{2\sigma^{2}}}},$$

where the approximate (because of the discrete nature of the distribution) mean and variance of G are $\bar{g}$ and $\sigma^{2}$, respectively. By knowing or assuming a valid value of the standard deviation σ (which could denote the variation in environmental, climate, or other conditions that affect reserve gain) depending on the geographical location and niche of a species, one would obtain a curve like the ones shown in Figure 6. In this case, reserves at the end of the current period depend upon the stochastic increment, so that Equation 8 is replaced by,

(11) $$r^{\prime }\left(r,r_{o},r_{i},g_{n}\right)=r-r_{o}-r_{i}+g_{n}.$$

The SDP equation (Clark & Mangel, 2000) would therefore be written as,

(12) $$W\left(r,t\right)=\sum_{n=0}^{N}p\left(g_{n}\right)\;\max_{r_{i},r_{o}}\left[\Phi \left(r_{o}\right)\cdot S_{\text{current}}\left(r_{i}\right)+S_{\text{future}}\left(r_{i}\right)\cdot W(r^{’}(r,r_{o},r_{i},g_{n},t+1),t+1)\right].$$

The left‐hand side of this above equation is the maximum accumulated lifetime reproductive success from time t onward, given that $R\left(t\right)=r$. The first term on the right‐hand side; the second term on the right‐hand side is future reproductive success t+1, which depends on the stochastic gain in reserves at $r^{\prime }\left(r,r_{o},r_{i},g_{n},t+1\right)$. The solution of this equation gives the optimal investment in reserves allocated toward parental effort for producing offspring $r_{o}^{*}\left(r,t,g_{n}\right)$ and in the parent's immune response $r_{i}^{*}\left(r,t,g_{n}\right)$ for each value of reserves, time, and food obtained at the end of the reproductive bout.

### 2.5 Forward Monte Carlo simulation algorithm

As in the deterministic case, the backward iteration provides a “handbook” of all optimal values of investment in offspring and survival for every reserve level, time, and gain in reserves. Unlike the deterministic case, because of the stochastic gain, more than one possible trajectory emerges going forward in time. To deal with this situation, we use Monte Carlo simulation of the dynamics of k=1,2,…K individuals, where initial reserves of individual k, $R_{k}\left(0\right)$ are determined by the Gaussian probability $p\left(g_{n}\right)$ in the stochastic case.

We follow each individual forward in time so that if individual k starts period t with reserve level r,

(13) $$R_{k}\left(t+1\right)=r+g_{n}-r_{o}^{*}\left(R_{k}\left(t\right),t,g_{n}\right)-r_{i}^{*}\left(R_{k}\left(t\right),t,g_{n}\right)\;\text{with probability}\;p\left(g_{n}\right).$$

**FIGURE 7:**
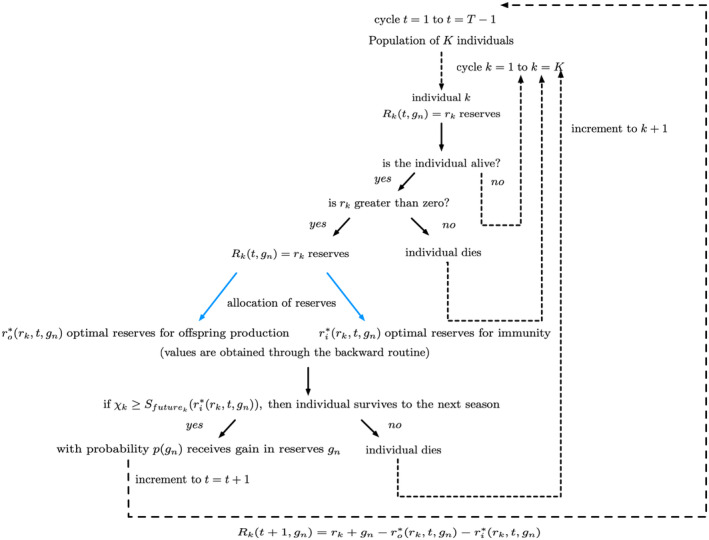
Forward stochastic routine. Here, $g_{n}$ denotes gain in reserves at the end reproductive period by an individual k, and $p\left(g_{n}\right)$ is the probability of receiving $g_{n}$ (as shown in Figure 6); and $S_{{\text{future}}_{k}}\left(r_{i}^{*}\left(r_{k},t,g_{n}\right)\right)=e^{\frac{-M}{1+\gamma r_{i}^{*}\left(r_{k},t,g_{n}\right)}}$ is the probability of survival to next reproductive season. The values $\chi_{k}\in \left[0,1\right]$ and $\rho_{k}\in \left[0,1\right]$ are two numbers drawn at random for each individual at every time which determine their survival to the next brooding season and gain in reserves, respectively.

Figure 7 shows a flowchart illustrating this algorithm.

## 3 RESULTS

In the deterministic case, we saw (Figure 4) that there is an effect of the relative time spent in parental investment (pregnancy or brooding) and background mortality on immunocompetence. Now, we explore the state dynamics and investment when the gain in reserves is stochastic, particularly: (1) the effect of parental investment on immunocompetence with time, (2) how the optimal allocation of reserves manifests in species with varying lifespan or varying natural background mortality, and (3) the emergence of realized mortality.

### 3.1 Effect of time spent in parental investment on immunocompetence

In Table 2, we show the effect of the fraction of time spent in pregnancy or brooding on investment in immunocompetence. The individuals of this population were tracked from the beginning until the time of reproductive senescence T. The value of $r_{i}$ increases with longer relative fraction of time between breeding events spent in pregnancy or brooding f (at the expense of direct investment in offspring) highlighting the effect of the fraction of time spent in parental investment on immunocompetence. This effect is more pronounced in species with shorter lifespans. We see for the three fraction of time f spent in parental investment cases, the immunocompetence initially increases from the initial time (as we choose an arbitrary value of $g_{n}$ at that time) to next time and then remains almost saturated from there. The saturation can be explained by the fact that an individual does not usually drastically get a big gain in reserves from one season to another.

**TABLE 2:** The optimal allocation of resources in short‐lived and long‐lived species is plotted in these figures.

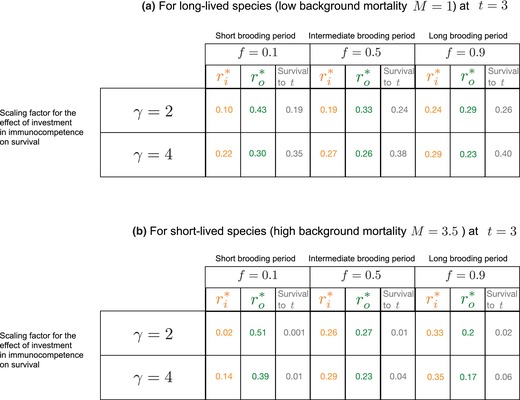

*Note*: Here, the value for background mortality is M=3.5 for short‐lived and M=1 for long‐lived species; a high and a low scaling factor for the effect of investment in immunocompetence $r_{i}$ on survival are represented by γ=2 and γ=4, respectively; short brooding period, intermediate brooding period, and long brooding period are denoted for f=0.1, f=0.5, and f=0.9, respectively; α=0.5 and $\Phi_{\max}=100$. The population size is 200. The results shown here for optimal values of $r_{i}^{*}$ (in orange) and $r_{o}^{*}$ (in green) are average values of the population of 200 individuals for σ=0.4 for the Gaussian probability distribution describing the gain in reserves as shown in Figure 6. The results do not change for a population size of a higher order. Thus, the results shown here are not due to the effect of population size.

### 3.2 Optimal allocation of reserves in long‐lived and short‐lived species

Without investment in immune competence, the lifespan is approximately 1/M (refer to the current and future survival functions in Table 1). Thus, long‐lived and short‐lived species will have low M and high M, respectively. In Table 2, we show reserve allocation toward immunocompetence in relation to f (relative fraction of time between breeding events spent in pregnancy or brooding), for both long and short‐lived species. The effect of σ (variation in high and low reserve gain between seasons) in the optimal allocation of reserves in short‐lived species do not differ much between the different values of σ (see Appendix S1). In Table 2, we show when γ is high, the fraction of alive individuals at every time is higher than when γ is low. A higher γ gives better effect of $r_{i}$ on survival. Hence, in the lower panel of Table 2 where γ=4, it pays more to invest in immunity when compared to γ=2. However, for low γ, there is relatively less effect from investing in $r_{i}$ compared to high lambda. Therefore, for both long‐lived and short‐lived species, fewer reserves are allocated toward offspring and more toward immunity when γ is high (as compared to when γ is low). For the same high value of γ, short‐lived species invest more toward their immunity than long‐lived species. As mentioned in the analysis that was calculated and plotted in the deterministic case (that we saw earlier), these individuals have a relatively low chance of surviving to a new breeding season. Hence, life‐history theory would make them invest as much as they can in the current reproduction. However, in our model, the reproductive success depends on the survival of the brooder though the period f. Thus, it pays to invest in the immune systems.

### 3.3 Effect of time spent in parental investment on realized mortality

**FIGURE 8:**
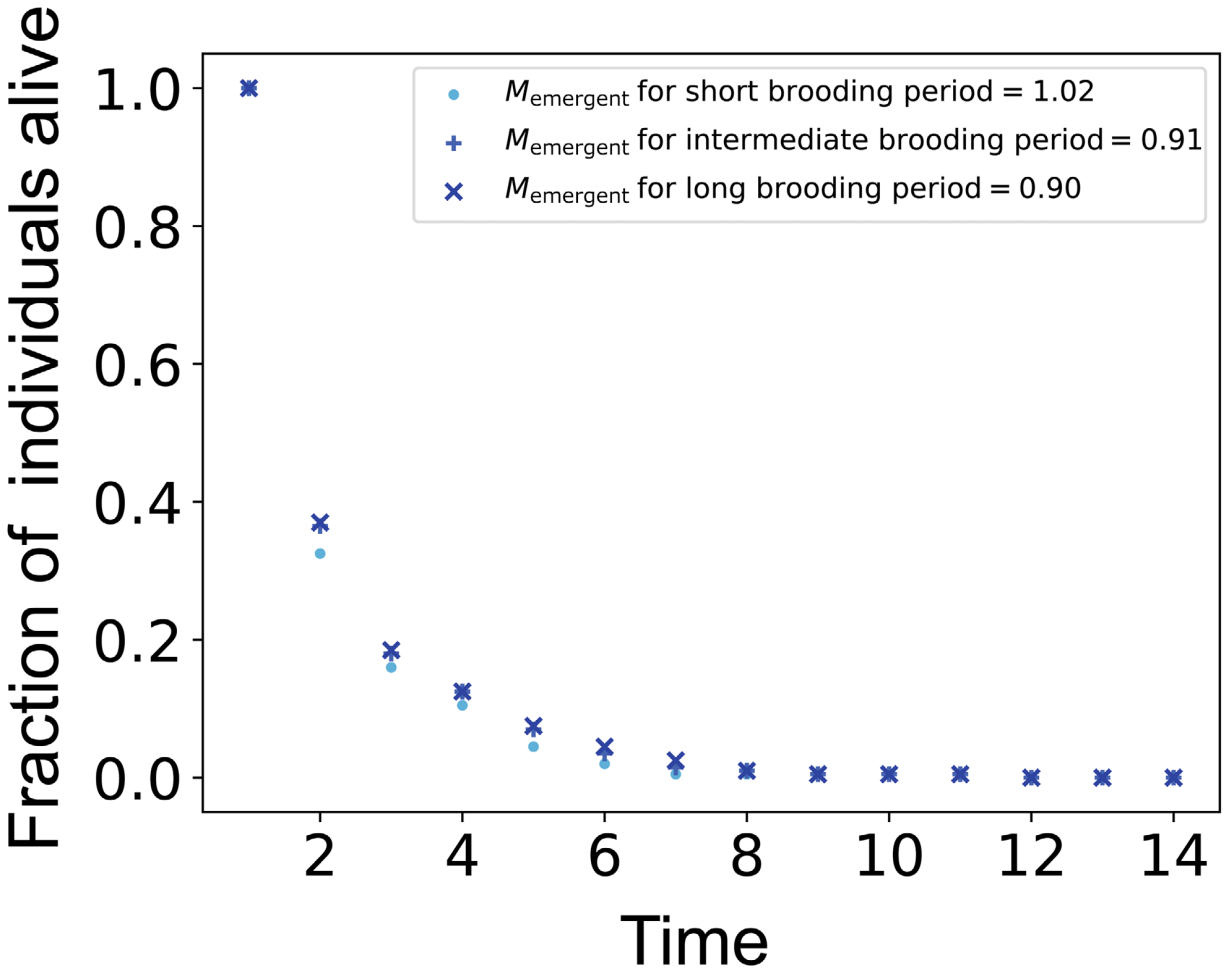
The effect of relative fraction of time between breeding events spent in pregnancy or brooding (f) on realized or emergent mortality is shown in these plots. The absolute value of the slope of the fitted curve is the emergent mortality or $M_{\text{emergent}}$. The values for background mortality and mortality scaling factor are M = 1.5 and γ=5, respectively. Here, short, intermediate, and long brooding periods correspond to f=0.1, f=0.5, and f=0.9, respectively. In all cases, emergent mortality is the result of investment in immune response and is less than 2/3 of background mortality and varies with brooding period.

Without investment in immune response, survival from one period to the next is $e^{-M}$. However, with investment survival from one period to the next depends on f and $r_{i}$. Thus, when optimal $r_{i}^{*}$ varies with f, it also affects the mortality. In this case, investment will let to an emergent mortality that can be computed as follows. We let $S_{\text{forward}}\left(t\right)$ denote the number of individuals surviving period t in the forward Monte Carlo simulation. We then determine the emergent mortality by setting $S_{\text{forward}}\left(t\right)=e^{-M_{\text{emergent}}\cdot t}$. In practice, $M_{\text{emergent}}$ is the the absolute value of the slope of $\log\left(S_{\text{forward}}\left(t\right)\right)$ versus time. In Figure 8, we show how the population numbers decrease with time and values of the emergent mortality.

However, this decrease is slower for a higher f, and this highlights the how pregnancy affects mortality that is when an individual undergoes pregnancy, it becomes beneficial to invest more reserves in immunocompetence leading to higher survival. Thus, our model shows that a longer relative fraction of time between breeding events spent in pregnancy or brooding (f) lead to more investment in the parent's immunocompetence $r_{i}$, and hence higher life expectancy.

## 4 DISCUSSION

In general, we predict that increased relative fraction of time between breeding events spent in pregnancy or brooding leads to an increase in the investment into the parent's immunocompetence. This is due to the effect of higher investment in immunocompetence ($r_{i}$) on survival, with a trade‐off with investment in offspring production ($r_{o}$). We showed that investment toward immunocompetence increases with increasing brooding period in both short‐lived and long‐lived species.

Our model also accounts for intraspecies scenarios where a higher value of f corresponds to the sex having long parental investment periods, and a negligible value of f refers to the other sex that makes a negligible investment. We could compare the investment of a parent having longer time spent in parental investment (high f) to another parent providing a negligible investment (f=0.1). This would correspond to different sexes of a species where one parent undergoes major parental investment such as pregnancy. Thus, these plots can also be used to highlight that the increase of investment in immunocompetence is higher for the parent having larger f. Thus, if a sex spends a long fraction of its time between breeding events in pregnancy or brooding compared to the sex that does not spend as much time in parental investment, we predict that this sex invests more toward its immunocompetence. That is, the evolution of sexual immune dimorphism can be driven by sex‐specific differences in parental investment, as demonstrated in empirical studies (Keller et al., 2017; Lin et al., 2016; Roth et al., 2011). Future experiments across a wide range of taxa focused on measuring immunocompetence between the sexes at various stages of their lifetime and concerning parental investment will shed more light on understanding sexual immune dimorphism across the animal kingdom. The studies by Foo et al. (2017) and Kelly et al. (2018) focus on the effect of hormones on sexual immune dimorphism and female bias in sexual immune dimorphism. However, in our model, the focus was the time spent in parental investment between brooding periods. Importantly, we also looked at cases such as seahorses and pipefishes where the major parental investment in done by males and have a male‐biased sexual immune dimorphism. We also studied the effect of differences in the time spent in parental investment between species on immunocompetence. Thus, our model is not limited to sexual immune dimorphism or intraspecies scenarios but can be applied to understand interspecies scenarios as well. Stoehr and Kokko (2006) and Medley (2002) showed that sex differences in parasitic impact might cause either sex to invest more into immunocompetence. Therefore, a potential enhancement to our model related to immunology could involve including host–pathogen dynamics. Currently, we capture the results of the host–pathogen dynamics with the parameter γ that describes the effect of investment in immunocompetence ($r_{i}$) on survival. Varying γ along with another parameter that describes either the pathogen impact in a particular environment or parameters that describe low and high infectious environments could tackle finer details and provide comprehensive results.

Different taxa perform diverse forms of parental investment, which most generally consists of three parts (initial gamete production, internal pregnancy, and external parental care) and varies between species and within the sexes of a species (Trivers, 1972). An important assumption in our model is that the survival of offspring is dependent on survival of the brooding/pregnant parent. Hence, we are not considering parental care in general, but rather more tightly linked relationships. Our model can be extended to the different parts of parental investment and ornamentation. It might be practical to study their effect on the immune response by varying one trait and keeping the others as constant values.

Informed by our theoretical predictions, further empirical studies that investigate the effect of parental investment on sexual immune dimorphism would be very useful. These studies would shed more light on how this effect is manifested in long‐ and short‐lived species and species with diverse reproductive strategies (semelparity or iteroparity). This could also provide us with a general understanding of how diverse sex‐specific life‐history traits affect each other.

## AUTHOR CONTRIBUTIONS

**Vandana Revathi Venkateswaran:** Conceptualization (equal); formal analysis (equal); investigation (equal); methodology (equal); project administration (equal); validation (equal); visualization (equal); writing – original draft (equal); writing – review and editing (equal). **Chaitanya S. Gokhale:** Funding acquisition (equal); investigation (equal); supervision (equal); validation (equal); visualization (equal); writing – original draft (equal); writing – review and editing (equal). **Marc Mangel:** Conceptualization (equal); investigation (equal); methodology (equal); project administration (equal); supervision (equal); validation (equal); visualization (equal); writing – original draft (equal); writing – review and editing (equal). **Sigrunn Eliassen:** Investigation (equal); methodology (equal); supervision (equal); validation (equal); visualization (equal); writing – original draft (equal); writing – review and editing (equal).

## CONFLICT OF INTEREST STATEMENT

The authors declare no conflicts of interest.

### OPEN RESEARCH BADGES

This article has earned an Open Materials badge for making publicly available the components of the research methodology needed to reproduce the reported procedure and analysis. All materials are available at https://github.com/abivandy/Dimorphism.

## Supporting information


Appendix S1

## Data Availability

There are no data to be archived.
