# Supplementary material for: Effects of time spent in pregnancy or brooding on immunocompetence
Source: Ecol Evol. 2024 Jan 4;14(1):e10764. doi: 10.1002/ece3.10764 (PMC10767163; doi:10.1002/ece3.10764)
Supplement: Supplementary file 1 — Appendix S1 [file ECE3-14-e10764-s001.zip › ece310764-sup-0001-AppendixS1.pdf]

Electronic Supplementary material

S.1 Effect of the scaling factor of optimal investment on survival and background mortality on the optimal investment towards immunocompetence in semelparous species

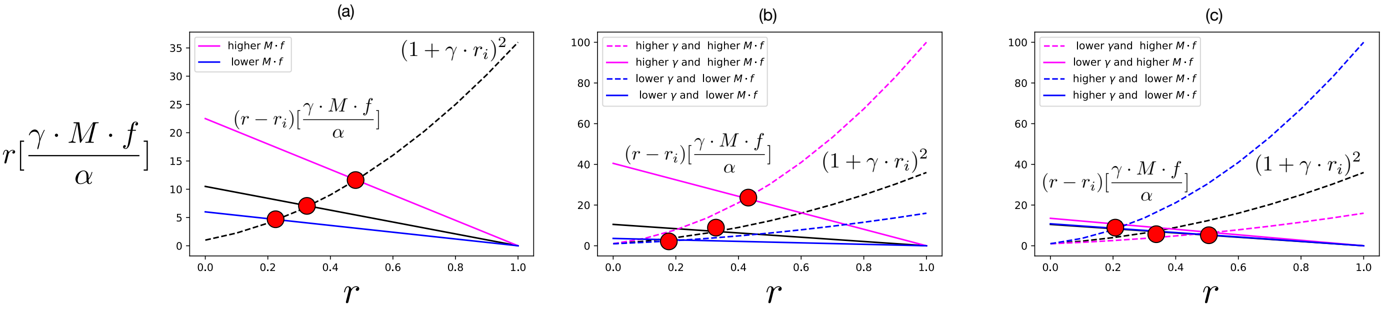

Figure S.1: The solutuion (r_i^* denoted by solid red circles) to equation S.4 for different cases is plotted here. Equation S.4 can be written as $(1 + \gamma \cdot r_i)^2 = (r - r_i)[\frac{\gamma \cdot M \cdot f}{\alpha}]$. (a) Case 1: Comparison of r_i^* between a reference value (black) of $M.f$ with a higher (pink) and a lower value (blue) of $M.f$ keeping every other parameter as constant. Here, we see as $M.f$ increases, r_i^* also increases. (b) Case 2: We see the increase in r_i^* when both γ and $M.f$ increase or decrease with respect to a reference $M.f$ and γ values. (c) case 3: The value of γ is made to increase or decrease as $M.f$ decreases or increases, respectively. In this case, we see some interesting dynamics: here, as $M.f$ increases, r_i^* decreases; and when $M.f$ decreases, r_i^* increases. The values for the reference line/curve (in black) used here are, $M = 1.5$, $f = 0.6$, $\gamma = 5$, and $\alpha = 0.4$.

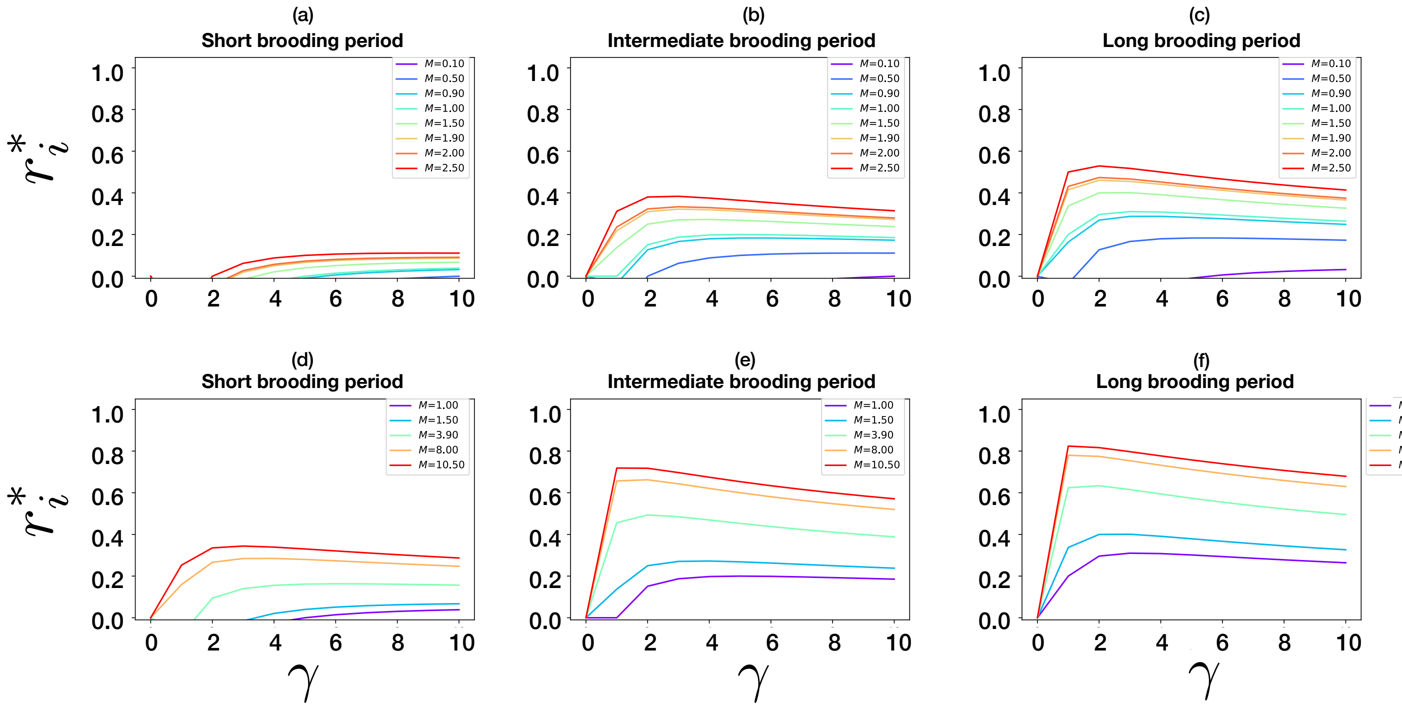

Figure S.2: To further understand the previous Figure S.1 in detail, we delve into how r_i^* changes with M and γ. Only non-negative values for r_i^* are valid because we are talking about resources here. Thus, we solve equation S.4 numerically considering the condition that $r_i^* \geq 0$, and we arrive at the inequality $M \cdot \gamma \geq 1$ given by equation S.10. Thus, we observe that $r_i^* = 0$, when $M \cdot \gamma = 1$. The solution $r_i^* \geq 0$ for $M = 0.1$ only when $\gamma \geq 10$, for $M = 0.5$ only when $\gamma \geq 2$, for $M = 1$ only when $\gamma \geq 1$, and so on, as shown here.

Here, we shall explore the effect of the scaling factor of optimal investment on survival, γ and the other parameters on r_i^*. Consider the reproductive fitness for a semelparous individual given by,

$$W(r|r_i) = \Phi_{max} \cdot (r - r_i)^{\alpha} \cdot e^{-\frac{M \cdot f}{1+\gamma \cdot r_i}}. \qquad \text{(S.1)}$$

taking log on both sides,

$$log(W(r|r_i)) = log(\Phi_{max}) + \alpha \cdot log(r - r_i) - \frac{M \cdot f}{1 + \gamma . r_i} \qquad \text{(S.2)}$$

Now,if we take the derivative and set it to zero, then we find the solution,

$$0 = \frac{-\alpha}{r - r_i} + \frac{\gamma \cdot M \cdot f}{(1 + \gamma \cdot r_i)^2} \tag{S.3}$$

or

$$(1 + \gamma \cdot r_i)^2 = (r - r_i)[\frac{\gamma \cdot M \cdot f}{\alpha}] \tag{S.4}$$

and this is plotted in Figure S.1. As shown in this Figure S.1, we see that when γ if fixed, and we vary M, then we see that as M rises, r_i^* also rises. However, when we vary both γ and M, then interesting dynamics emerge. When M and γ both increase or decrease, then r_i^* decreases or increases, respectively. But, when M increases and γ decreases or M decreases and γ increases, then we apparently see a reverse effect, i.e. r_i^* decreases when γ drops and increases when γ rises. Since γ scales the effect of investment in immunocompetence (r_i) on survival, a high value of γ would mean that the individual has a good immune defense because it can increase survival with a small investment, while a small value of γ implies that significant investment is needed to improve survival. So one would assume that with increasing (or decreasing) γ, an individual's optimal allocation to immunocompetence (r_i^*) should always decrease (or increase). But, through this analysis, we find that this can happen only when both the background mortality M and γ increase (or decrease) together.

To prove this, we actually solve the Equation S.4 numerically, instead of just looking at it graphically. We write this equation in its quadratic form as,

$$\gamma^2 \cdot r_i^2 + [\gamma(2 + k)]r_i + [1 - (r \cdot k \cdot \gamma)] = 0. \tag{S.5}$$

where $k = \frac{\cdot M \cdot f}{\alpha}$.

The solution to this equation will be,

$$r_i^* = \frac{-[\gamma(2+k)] \pm \sqrt{([\gamma(2+k)]^2 - 4 \cdot \gamma^2 \cdot [1 - (r \cdot k \cdot \gamma)])}}{2 \cdot \gamma^2}. \tag{S.6}$$

which can be simplified into,

$$r_i^* = \frac{-(2+k) \pm \sqrt{((2+k)^2 - 4 \cdot (1 - (r \cdot k \cdot \gamma)))}}{2 \cdot \gamma}. \tag{S.7}$$

For $r_i^* \geq 0$, we need to find the conditions that make $r_i^* \frac{-(2+k)+\sqrt{((2+k)^2-4\cdot(1-(r\cdot k\cdot\gamma)))}}{2\cdot\gamma} \geq 0$ (since $r_i^* = \frac{-(2+k)-\sqrt{((2+k)^2-4.(1-(r\cdot k\cdot\gamma)))}}{2\cdot\gamma} < 0$ always).

For this to be finite and positive, the denominator should have $\gamma > 0$, and the numerator should be,

$$\sqrt{((2+k)^2 - 4.(1 - (r \cdot k \cdot \gamma)))} \geq (2+k). \tag{S.8}$$

This gives the inequality,

$$r \cdot [\frac{M \cdot f \cdot \gamma}{\alpha}] \geq 1. \tag{S.9}$$

When $r = 1$, and say, for instance, $f = 0.5$ (to look at the case of intermediate brooding period like we show in Table 2 in the main text), and $\alpha = 0.5$ as in all the figures in the main text, then,

$$M \cdot \gamma \geq 1. \tag{S.10}$$

or

$$M \geq \frac{1}{\gamma}. \tag{S.11}$$

Thus, we observe that $r_i^* = 0$, when $M \cdot \gamma = 1$. The solution $r_i^* \geq 0$ for $M = 0.1$ only when $\gamma \geq 10$, for $M = 0.5$ only when $\gamma \geq 2$, for $M = 1$ only when $\gamma \geq 1$, and so on, as shown in the Figure S.2(b). In Figure S.2, we plot a wider range of these parameters.
