# Supplementary figures and images for: Effects of time spent in pregnancy or brooding on immunocompetence
Source: Ecol Evol. 2024 Jan 4;14(1):e10764. doi: 10.1002/ece3.10764 (PMC10767163; doi:10.1002/ece3.10764)

$$r \left[ \frac{\gamma \cdot M \cdot f}{\alpha} \right]$$

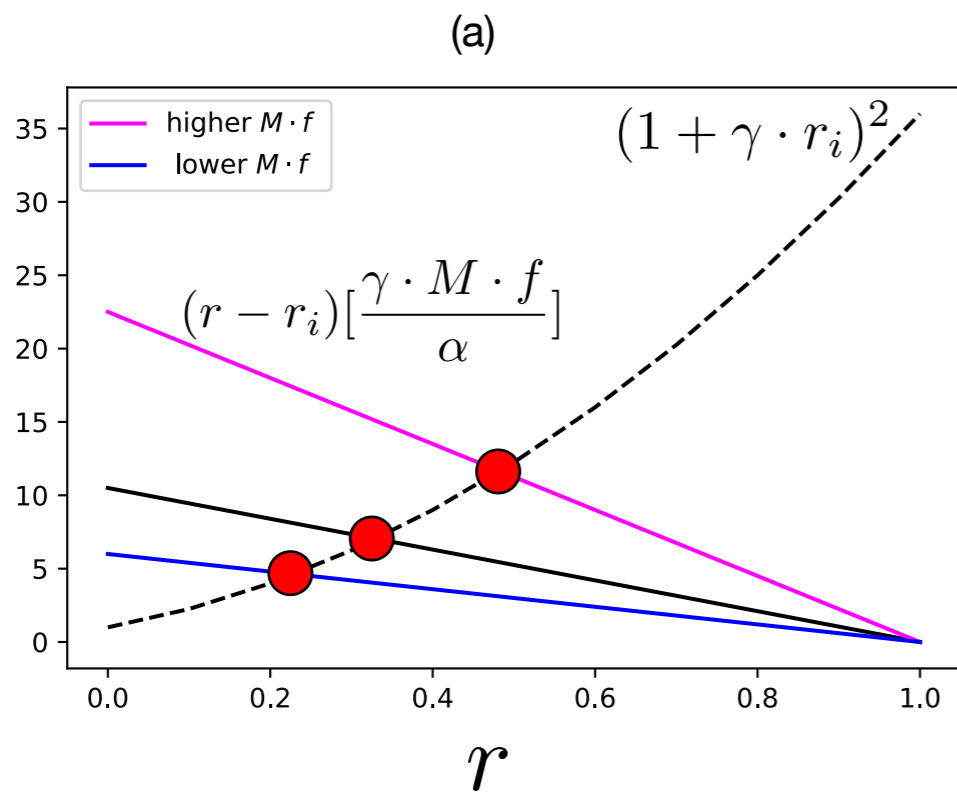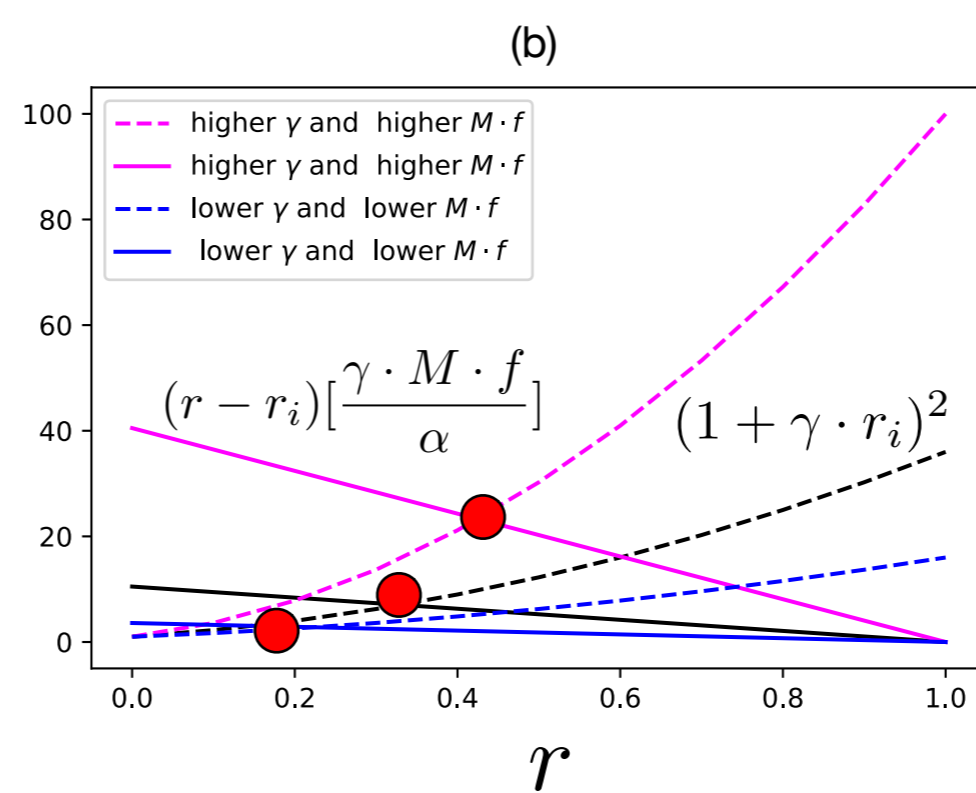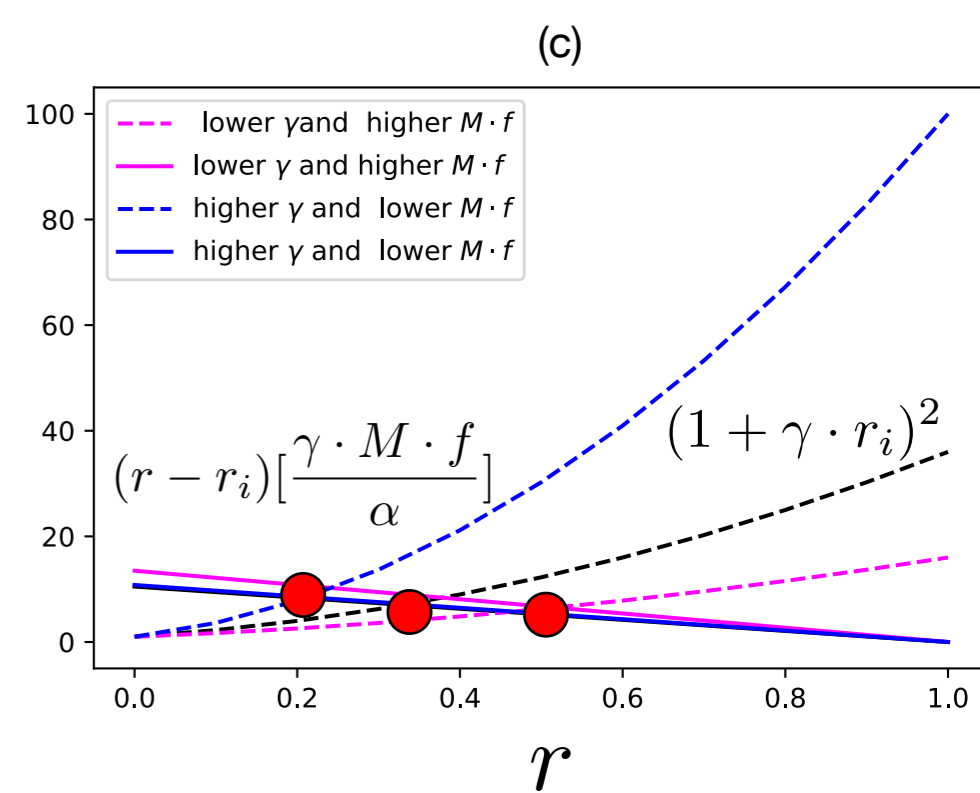

Supplement: Supplementary file 1 — Appendix S1 [file ECE3-14-e10764-s001.zip › Figure S1.pdf]

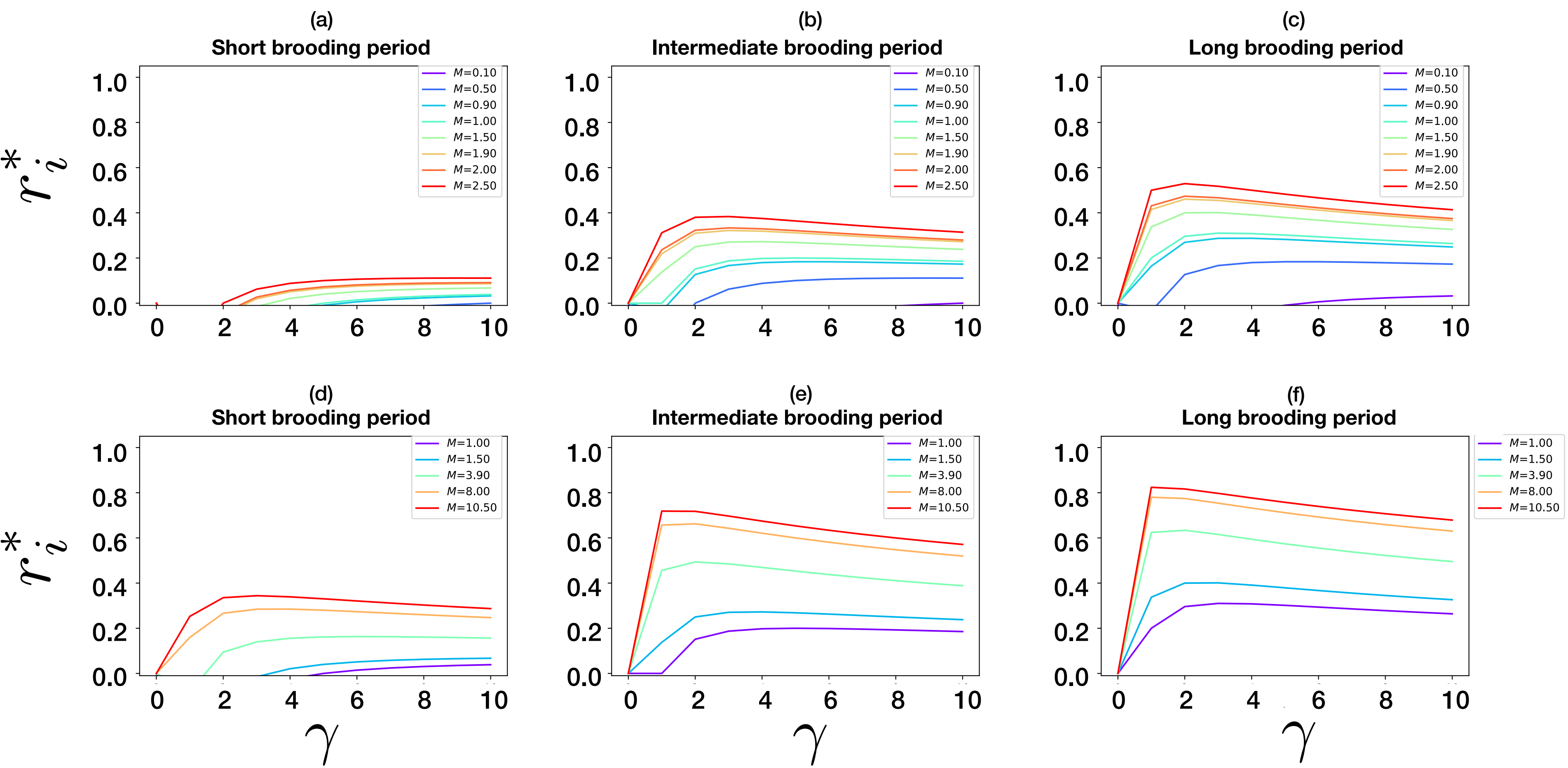

Supplement: Supplementary file 1 — Appendix S1 [file ECE3-14-e10764-s001.zip › Figure S2.pdf]
